# Single-Cell Sequencing Reveals Necroptosis-Related Prognostic Genes of Glioblastoma

**DOI:** 10.1155/2023/2926655

**Published:** 2023-02-20

**Authors:** Ping Zheng, Dabin Ren, Yu Cong, Xiaoxue Zhang, Yisong Zhang

**Affiliations:** ^1^Department of Neurosurgery, Shanghai Pudong New Area People's Hospital, Shanghai, China; ^2^Key Molecular Lab, Shanghai Pudong New Area People's Hospital, Shanghai, China

## Abstract

**Background:**

Glioblastoma (GBM) is one of the most malignant forms of brain cancer, with the extremely lower survival rate. Necroptosis (NCPS) is also one of the most wide types of cell death, and its clinical importance in GBM is not clear.

**Methods:**

We first identified necroptotic genes in GBM by single-cell RNA sequencing analysis of our surgical samples and weighted coexpression network analysis (WGNCA) from TCGA GBM data. The cox regression model with least absolute shrinkage and selection operator (LASSO) was used to construct the risk model. Then, KM plot and reactive operation curve (ROC) analysis were used to assess the prediction ability of the model. At last, the infiltrated immune cells and gene mutation profiling were investigated between the high- and low-NCPS groups as well.

**Result:**

The risk model including ten necroptosis-related genes was identified as an independent risk factor for the outcome. In addition, we found that the risk model is correlated with the infiltrated immune cells and tumor mutation burden in GBM. NDUFB2 is identified to be a risk gene in GBM with bioinformatical analysis and in vitro experiment validation.

**Conclusion:**

This risk model of necroptosis-related genes might provide clinical evidence for GBM interventions.

## 1. Introduction

Glioma is a common malignant brain tumor, and the disease rate is increasing each year [1, 2]. Among them, GBM is the most malignant type which holds about 70-80% of adult glioma patients [2], which is very likely to metastasize [3]. The treatment strategy for GBM is surgical resection. About half of GBM patients undergo a recurrence after brain surgery [4], even if they are combined with target therapy and cell therapy; however, the overall survival status of GBM is still very poor [5], which indicated that the molecular mechanism of GBM needed to be further explored.

Immune checkpoint inhibitor (ICI) therapy, such as anti-programmed cell death protein- (PD-) 1 or cytotoxic T lymphocyte-associated antigen-4 (CTLA-4) inhibitors, which have made breakthroughs in solid tumors with a higher TMB [6, 7], is in clinical trial in GBM [8, 9]. Nevertheless, few GBM patients do respond to ICIs, which might be due to the lower TMB in GBM [10]. Thereafter, it is essential to locate novel biomarkers of GBM.

Resistance to apoptosis is one of markers of tumor [11]. Oxidative phosphorylation and metabolic reprogramming are all involved in tumor resistance. Necroptosis is a new type of cell death [12], which has been reported in prostate cancer [13] and hepatocellular carcinoma [14]. In addition, necroptosis belongs to the immunogenic cell death and has a critical effect in TME [15]. Changes in the TME triggered by necroptosis are also involved in the ICI treatment [16]. However, almost no studies have investigated the role of necroptosis in glioblastoma.

So, it is critical to investigate the effect of necroptosis in GBM, and we used both TCGA GBM data and GSE 43378. We analyzed our own single-cell sequencing sample and performed combined analysis with TCGA GBM data. After identifying the necroptosis-related genes in GBM and dividing these data into the high- and low-NCPS group, we evaluated the different predictions between them. Furthermore, we investigated the immune infiltration signature and tumor mutation burden as well. Few studies have explored the necroptosis-related genes in GBM and the function from different cell clusters. Therefore, our findings might provide clinical evidence for GBM.

## 2. Methods

### 2.1. TCGA Data

GBM data was downloaded from TCGA on May 31st, 2022. clinical information is available for 169 GBM patients and five healthy controls.

### 2.2. Single-Cell Sequencing Data Obtainment and Processing

The scRNA-seq data were obtained from our surgical samples: one is glioblastoma, and the other is meningioma (serves as controls). Then, we carried out quality control and cells with <10% mitochondrial genes were captured, and gene numbers ranged from 200 to 10000 and in at least three cells. We integrated all samples via SCT correction. Next, uMAP method was used for the dimension reduction. We integrated both samples via SCT correction. Then, both tSNE and uMAP methods were used to reduce the dimension of data.

### 2.3. The Acquisition of Necroptosis-Related Genes

571 genes related to necroptosis were obtained from the GeneCards website. And a total of 68 necroptosis-related genes were found with a correlation efficiency > 1.

### 2.4. Gene Set Enrichment and Pathway Analysis

Here, ssGSEA is used to calculate the necroptosis score in each GBM patient. In addition, both UCell and irGSEA were used to assess the pathway enrichment in different cell clusters.

### 2.5. Weighted Coexpression Network Analysis (WGCNA)

WGCNA was carried out to identify the gene modules mostly correlated with necroptosis score in GBM.

### 2.6. Construction of the Risk Model Associated with Necroptosis

We had GBM patients with survival period from two to 3881 days. Both univariate and multiple Cox models were applied to evaluate the relationship between NCPS and the survival status of GBM patient. Risk score = coefficient of mRNA × risk genes. The LASSO is used to locate the survival-related genes. The “survminer” package was used to compare the survival status between the high-and low-risk NCPS groups.

### 2.7. External Validation of the Model

GSE 43378 was used as an external validation cohort for the risk model. NCPS of every patient was calculated in TCGA dataset and validated in the GSE dataset. Three-dimension principal component analysis (3D-PCA) was applied to assess the classification of GBM patients. We also used the Chinese Glioma Genome Atlas (CGGA) data to validate the expression and predictive value of prognosis-related necroptotic genes.

### 2.8. Correlation Analysis of Immune Infiltration and Mutation Profiling

We then explored the correlation between NCPS and infiltrated immune cells, with Quantiseq algorithm. We used the Wilcoxon test to compare the quantification of infiltrated immune cells between the high- and low-NCPS groups. The association between risk scores and the immune-checkpoint inhibitor (ICI) markers was also explored by the Wilcoxon test. Then, we investigated the difference of tumor-infiltrating cells (TILs) and repair genes between different NCPS groups. Finally, we calculated the tumor mutation burden (TMB) between the two groups and listed the top 20 mutation genes between groups.

### 2.9. The Construction of a Nomogram

A nomogram constructed in this study was to evaluate the mortality rate in GBM by combining NCPS risks and clinical characteristics of this model. The accuracy of the nomogram was also assessed by both prognostic ROC and DCA curves as well.

### 2.10. Cell Lines and Cell Culture

The U87 human glioma cell lines were obtained from the Typical Culture Preservation Commission Cell Bank, Chinese Academy of Sciences. U87 was maintained on gelatinized 10 cm plate in RPMI-1640 medium (Asia-Vector Biotechnology, Shanghai) supplemented with 10% FBS (fetal bovine serum) (Gibco, NYU, USA), 100 U penicillin/100 mg streptomycin (Gibco, NYU, USA) at 37°C, and 5% CO_2_.

### 2.11. Silencing of NDUFB2 by Transient Transfection siRNA

The siRNA sequences against human NDUFB2 and nonsilencing were designed and chemically synthesized by Asia-Vector Biotechnology (Shanghai, China). U87 cells were transfected with 100-200 pmol siRNA using 5-10 *μ*L INTERFERin® transfection reagent (Polyplus) in a 6-well plate. After 24-48 hours, cells were collected to perform cell proliferation and colony formation.

### 2.12. Overexpression of NDUFB2

To overexpress NDUFB2, the human NDUFB2 coding sequence (CDS) was prepared by reverse transcription polymerase chain reaction (RT-PCR) from normal colorectal tissues and cloned into the pLVX-IRES-mCherry vector at Xba I and BamH I sites for overexpressing NDUFB2 inU87 cells. Lentiviruses were packaged and produced in 293T cells. The supernatant of virus production was collected and filter-sterilized to infect the corresponding cells. The stably transfected cells were sorted and collected. The sequence for overexpression of NDUFB2 (OE) is as follows:

*NDUFB2-F*: 5′-CTCGGATCCGCCACCATGTCCGCTCTGACTCGGCT-3′

*NDUFB2-R*: 5′-CCCTCTAGACTCGAGGTCTTCATCATCAGGAGGGA-3

### 2.13. CCK-8 Assay

Each group of cells was adjusted to 1000 cells per well. Then, 10 *μ*L of CCK-8 solution (Beyotime Biotechnology, Haimen, China) was added to the cell dish after 24 hours. The blank control had only CCK-8 solution. The absorbance (OD) value of each well was read at 490 nm every 24 hours for 3 days.

## 3. Results

**Figure 1:**
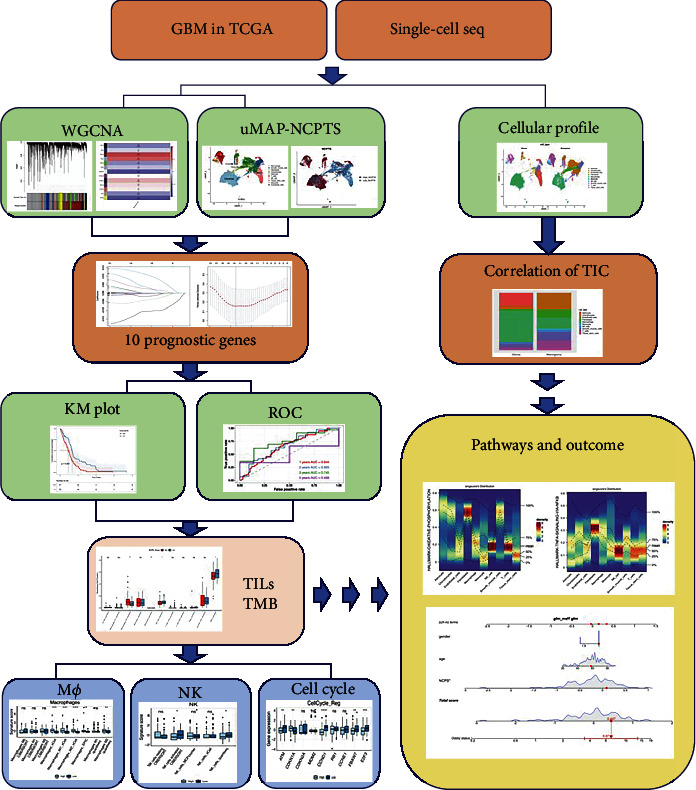
A schematic flow of the study. We first did the WGCNA from TCGA GBM data and obtained the prognosis-related gene modules, which are intersected with the NCPTs to get the prognosis-related necroptosis genes in GBM. Then, we applied KM plot to assess the association between the survival status, immune infiltration, and risk factors, which is further validated in the in-house single-cell RNA sequencing data (right). The nomogram was established based on the NCPTs, and associated gene enrichment and pathways were analyzed accordingly.

A schematic flow of our study is shown in Figure 1.

### 3.1. Single-Cell Sequencing Data Analysis

**Figure 2:**
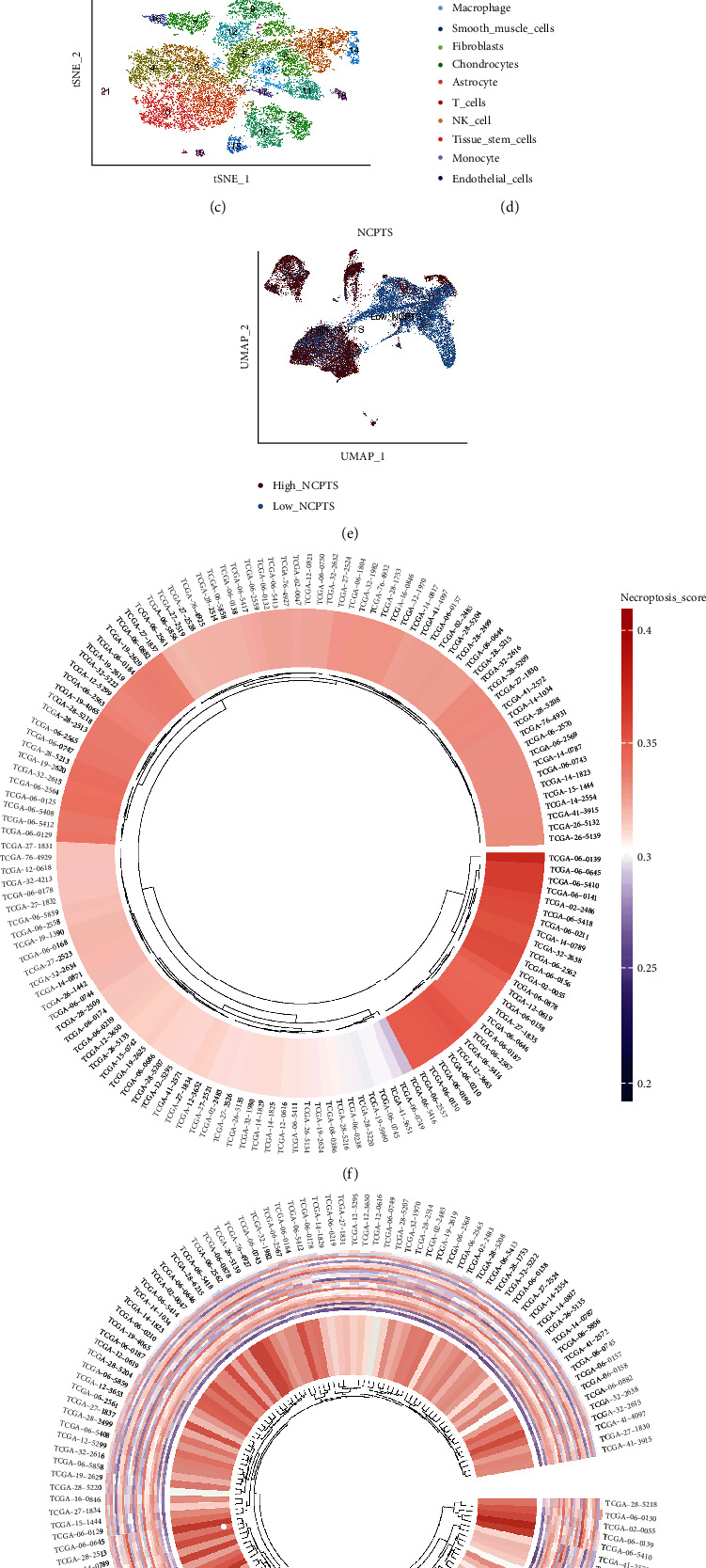
Single-cell sequencing analysis of glioma and meningioma sample. (a, b) 23 cell cluster dimensionality reduction with uMAP and cluster analysis. (c, d) Dimensionality reduction with tSNE and cluster analysis. (e) The cells were divided into high- and low-necroptosis groups. (f) Circos figures show the distribution of high and low necroptotic groups in TCGA samples. Red indicates higher NCPS, and blue indicates the lower NCPS. (g) ssGSEA together with heat map show the distribution of high- and low-NCPS samples in TCGA. (h, i) WGCNA found that MEbrown, MEcyan, MElightcyan, and MEyellow modules were closely related to the necroptosis score.

We first applied the single-cell sequencing in our GBM and meningioma (serves as controls) surgical samples. As shown in Figure 2, cells were clustered into 23 clusters by the uMAP and tSNE method (Figures 2(a)–2(d)). Then, 68 genes related to necroptosis were added with the “AddModuleScore” function. Then, the distribution of necroptosis genes in each cell cluster was showed by uMAP (Figure 2(e)). According to the Human Primary Cell Atlas Data, we clustered cells into 10 clusters with the Single R package. Several cell clusters such as: macrophage, smooth muscle cells, fibroblasts, chondrocytes, astrocytes, T cells, NK cells, tissue stem cells, monocyte, and endothelial cells were identified, respectively. (Figures 2(a)–2(d)). We found that higher NCPTS were distributed in macrophages, NK, and T cells, while lower NCPTS in astrocytes and fibroblasts and 676 genes were identified between higher and lower NCPTS groups (Figure 2(e)). As the NCPTS seems to be related with the immune cell distribution, we further classified TCGA samples with medium NCPTS as well (Figure 2(f)) and grouped them together with ssGSEA score (Figure 2(g)). The differential-expressed genes (DEGs) between the high- and low-NCPTS groups are listed in the supplementary file (available here) (scRNA_diffsheet).

### 3.2. WGCNA

WGCNA was used to identify the gene modules related to necroptosis in 143 TCGA GBM samples. By setting the soft threshold at 12, the minimum number of module genes at 100, and the similarity threshold lower than 0.25, a total number of 12 nongray gene modules were identified (Figure 2(h)). As shown in Figure 2(i), we identified that MEbrown, MEcyan, MElightcyan and MEyellow were statistically associated with NCPTS. The module genes are attached in the supplementary file (module_data). According to the correlation value > 0.4, genes from above modules were selected for further analysis.

### 3.3. Construction and Validation of a Necroptosis-Related Prognostic Model

**Figure 3:**
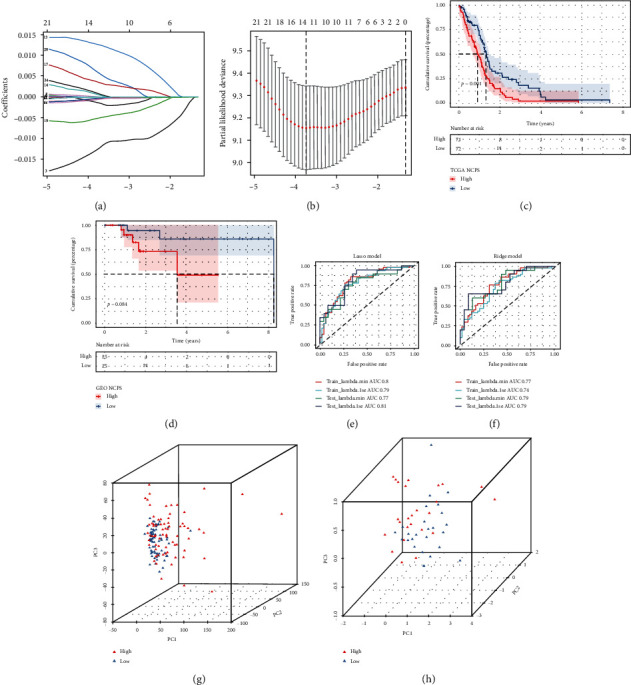
Construction of a necroptosis-related risk model. (a, b) Ten necroptosis-related genes were screened to establish the risk model. (c) The survival analysis between the high- and low- risk groups in TCGA cohort. (d) The survival analysis between the high- and low- risk groups in the GEO cohort. (e, f) ROC curve of TCGA and GEO cohort. (g) The PCA between the high- and low- risk groups in TCGA cohort. (h) The PCA between the high- and low- risk groups in the GSE 43378 cohort.

First, we obtained 313 intersected genes between sc-seq data and WGCNA genes related to necroptosis, which were further identified with COX and LASSO analysis to the prognosis of patients (COX genes are listed in the supplementary file, Unicox_rex sheet). 10 genes were finally obtained, as the gene contraction curve tended to be stable (Figures 3(a) and 3(b)). The expression profile of these 10 genes is listed in the supplementary file (TCGA_NCPS). Then, the prognostic model was calculated as follows: NCPS = ENAH^∗^ (−0.01060161) + EEF1B2^∗^(−1.29372811) + RPS18^∗^(−2.63853005) + NDUFB2^∗^(0.0109492) + ARL3^∗^(−0.00194365) + RNH1^∗^(0.00264256) + C12orf57^∗^(−0.00421293) + RPL13^∗^(−5.68893971) + RPS19^∗^(−3.37923540) + SELENOM^∗^(0.00355917).

Figure 3(c) shows that the higher NCPS group had a poorer prognosis in TCGA GBM (*P* = 0.001). However, in the GSE 43378 validation cohort, we also found that patients with high-NCPS had a worse prognosis as well but did not reach the statistical difference (*P* = 0.084, Figure 3(d)). And this may be due to the smaller sample size in the GEO dataset. In order to assess the predictive ability of NCPS in the assessment of the outcome in GBM, ROC analysis was performed.

As shown in Figures 3(e) and 3(f), AUCs in both LASSO and ridge models were more than 0.7, which suggests that NCPS can predict the GBM outcomes highly. At last, 10 LASSO genes were included in the PCA in TCGA and validated GEO dataset, respectively, and it was shown that this NCPS model could classify patients well into higher and lower groups (Figures 3(g) and 3(h)).

### 3.4. Immune Infiltration Analysis and Mutation Landscape

**Figure 4:**
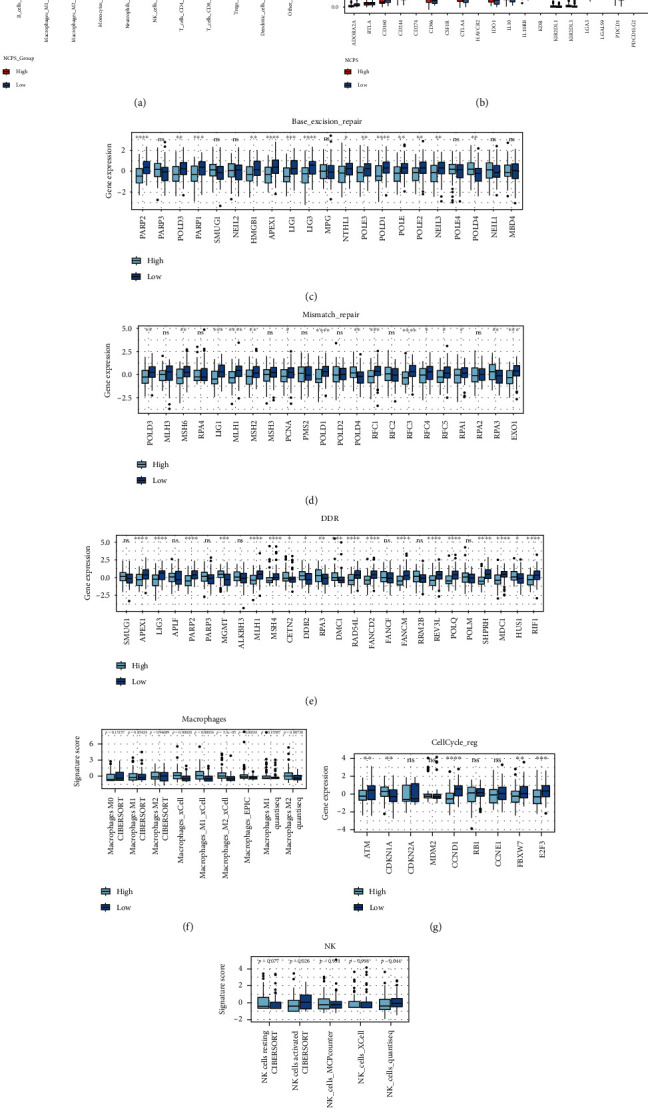
Immune infiltration analysis related to NCPTS in TCGA GBM. (a) Expression of infiltrated immune cell between the high- and low-NCPS groups by Quantiseq analysis. (b) Relative expression of immune checkpoint genes between the high- and low -NCPS groups. The results showed that the expression trend of ICI-related genes was lower in the high-NCPS group. (c–e) The relative expression of base excision repair, mismatch repair, and DDR genes between the high- and low-NCPS groups and the expression trend of DNA repair-related genes were lower in the high-NCPS group. (f) The relative number of infiltrated macrophages between the high- and low-NCPS groups. (g) The expression trend of cell cycle-related genes between the high- and low-NCPS groups. (h) The relative number of infiltrated NK cells between the high- and low-NCPS groups.

As GBM is generally resistant to the immune therapy, we further investigated the tumor-infiltrated lymphocytes (TILs) and microenvironment between NCPS subgroups. We found that there were more macrophage M2 cells and less NK cells in the high-NCPS group as demonstrated in Figure 4(a). Then, the expression of ICI markers was compared between the different groups. As demonstrated in Figure 4(b), most of the ICI-related genes, such as ADORA2A, KDR, and LAG3, were found to be decreased in the high-NCPS group. Accordingly, the base excision repair, mismatch repair, and DDR genes were much lower in the high-NCPS group compared to the low-NCPS group (Figures 4(c)–4(h)). Meanwhile, the increased infiltrated macrophages and fewer cell cycle genes and NK cells were found in the high-NCPS group as well. We found that the high-NCPS group was related to the lower immune infiltration and tumor mutation burden (TMB).

**Figure 5:**
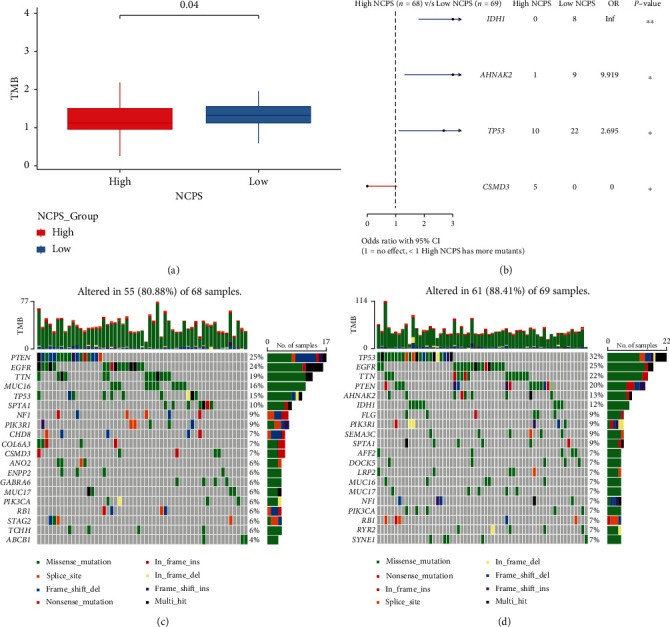
Mutation landscape analysis related to NCPTS in TCGA GBM. (a) The difference of TMB between the high- and low-NCPS groups. (b) The difference of top mutation genes between the high- and low-NCPS groups. (c, d) Mutation landscape between the high- and low-NCPS groups of TCGA dataset. The top 20 mutation genes between the two groups are listed on the left side, and the percentage of each mutation is listed on the right side. The mutation incidence rate of top 3 genes PTEN, EGFR, and TTN in the high risk groups was 25% (17 of 68 samples), 24% (16 of 68 samples), and 19% (13of 68 samples) of GBM patients, while, in the low risk groups, the mutation incidence rate of TP53, EGFR, and TTN was 32% (22of 69 samples), 25% (17 of 69 samples), and 22% (15 of 69 samples).

Then, we investigated the landscape of top twenty mutated genes between the high- and low-NCPS group. First, we calculated the TMB value between the high- and low-NCPS group and found that the high-NCPS group had lower TMB (Figure 5(a)). Representatively, CSMD3 mutation occurred in the high-NCPS group and IDH1, AHNAK2, and TP53 mutations were found in the lower NCPS group (Figure 5(b)). In addition, the top 20 mutated genes are listed in Figures 5(c) and 5(d). The top 3 mutated genes in the high-NCPS group were PTEN, EGFR, and TTN, while the ones in the low-NCPS group were TP53, EGFR, and TTN (Figures 5(c) and 5(d)).

### 3.5. Specific Cell Location of Hub Genes

**Figure 6:**
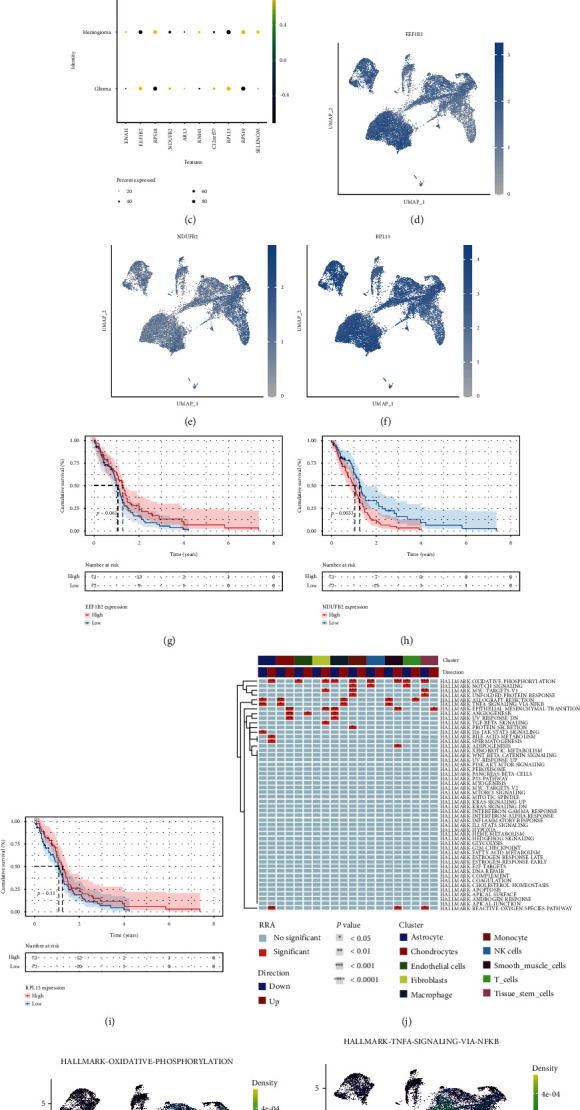
Single-cell sequencing analysis to explore the cell localization of modeling genes. (a) The cell cluster distribution between glioma and meningioma. (b) The cellular percentage between glioma and meningioma. (c) The expression of modeling genes between meningioma and glioma. (d–f) The cellular distribution of EEF1B2, NDUFB2, and RPL13. (g–i) Survival analysis of EEF1B2, NDUFB2, and RPL13 in GBM. GBM patients with high NDUFB2 level had worse outcome than those with low NDUFB2 level (*P* = 0.0033). (j) Different functions in different cell clusters revealed by UCell and irGSEA. (k, l) Top enrichment pathway: oxidative phosphorylation and inflammation pathway.

We analyzed the scRNA-seq data to explore the location of hub genes. As demonstrated in Figures 6(a) and 6(b), we found that there were more macrophages and less NK cells in the glioma compared to the meningioma group. This is quite consistent with our immune infiltration analysis (Figure 6(c)). As EEF1B2, NDUFB2, and RPL13 were more obvious in the glioma group, we further showed them in the cell clusters (Figures 6(d)–6(f)). EEF1B2 was dominantly found in macrophage cells, NDUFB2 was dominantly found in astrocytes, and RPL13 was widely distributed in all cells as this is a ribosome gene. In addition, we conducted survival analysis on these three genes. Our findings demonstrate that only patients with high expression of NDUFB2 had a poorer prognosis (*P* < 0.001, Figures 6(g)–6(i)).

To further investigate the molecular mechanisms of different cell types, we applied the UCell and irGSEA and found that different cell clusters carried out different functions in the heat map (Figure 6(j)). Therefore, we chose the top enrichment pathway: oxidative phosphorylation and inflammation pathway. We found that the high NCPS had a lower oxidative phosphorylation enrichment (Figure 6(k)) and higher TNFA and NFKB inflammation extent (Figure 6(l)).

### 3.6. Nomogram Construction

**Figure 7:**
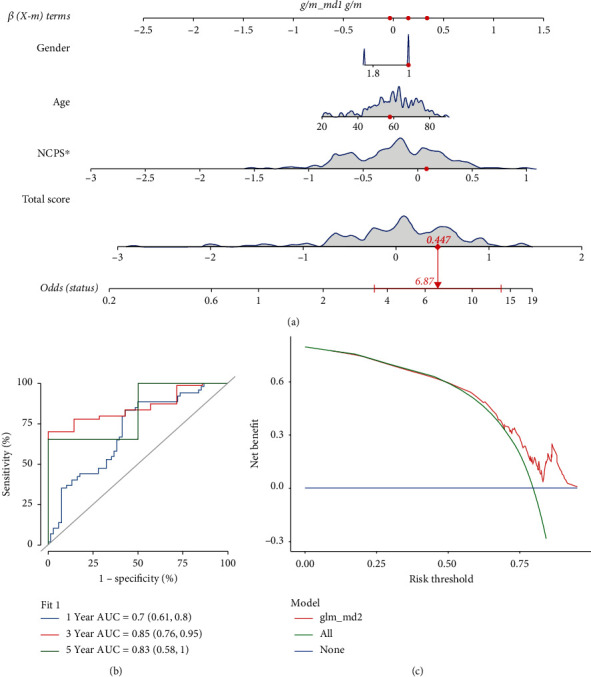
The construction of a nomogram. (a) Nomogram of TCGA patient; the mortality rate of 1, 3, and 5 years was estimated to be 0.496, 0.979, and 0.999. (b) The prognostic ROC analysis showed that AUCs in 1, 3, and 5 years were 0.7 (0.61-0.8), 0.85 (0.76-0.95), and 0.83 (0.58-1), respectively. (c) DCA indicated that the role of the nomogram was better than other clinical features in GBM patients.

To better evaluate the risk factors in GBM, a nomogram was drawn based on combined clinical features and NCPS. As demonstrated in Figure 7(a), the gender, age, and NCPS score of one representative patient and the mortality rate of the patient in 1, 3, and 5 years were estimated to be 0.496, 0.979, and 0.999. To further assess the prediction ability of the nomogram, we performed the ROC analysis. Our findings suggest that the AUCs in one, three, and five years were 0.7 (0.61-0.8), 0.85 (0.76-0.95), and 0.83 (0.58-1), respectively (Figure 7(b)). We carried out the decision curve analysis (DCA) as well and found that the predicting ability of the nomogram was better than other clinical features, indicating that the NCPS nomogram has a good predicting ability for GBM (Figure 7(c)).

### 3.7. Validation of the Independent Risk Gene NDUFB2 in CGGA

To validate the expression and prediction value of NDUFB2 in the Chinese Glioma Genome Atlas (CGGA), we first explored the expression of NDUFB2 in different grades of glioma and we found that it was increased with the higher degree of glioma (Supp Fig 1A); meanwhile, the expression of NDUFB2 is much lower in the IDH-1 mutant type compared to IDH-1 wild-type groups (Supp Fig 1B). In addition, as for subtypes of CGGA, we found that the expression of NDUFB2 was much higher in the classical group (Supp Fig 1C). The AUC of ROC in predicting NDUFB2 for CGGA subtype (classical) is 0.646 (Supp Fig 1D). Again, NDUFB2 also has a tight connection with NDUFA2 and NDUFA13 (Supp Fig 1E). The heat map shows higher expression of NDUFB2 list in the high-grade glioma, classical subtype, and IDH-1 wild-type groups (Supp Fig 1F), and the trend is similar is other related genes. Furthermore, as for the survival status, consistently, we found that the expression of NDUFB2 is negatively associated with the survival status in glioma (*P* = 0.0084, Supp Fig 1G).

The main function of NDUFB2 is further explored by a web tool and showed that it is related to DNA repair, stemness, and cell cycle, and the role of NDUFB2 might be different in low-grade glioma (LGG) and GBM (Supp Fig 2A-C). Therefore, we further investigated the role of NDUFB2 with in vitro experiments. First, we verified that NDUFB2 promoted cellular proliferation of U87 cells with CCK-8 assay (Supp Fig 3A). Moreover, the expression of NDUFB2 promoted tumorigenesis with clonal formation (Supp Fig 3B).

## 4. Discussion

Although glioma and meningioma both originate in the central nervous system (CNS), a series of different biological features and therapeutic responses are involved [17], such as ICIs, which have achieved greatness in solid tumors. However, the role of immunotherapy is not satisfactory in CNS tumors, especially in GBM [18]. Mechanisms such as lower TMB and metabolic reprogramming are considered to be a weak response of GBM to immunotherapy [19].

Necroptosis is an inflammatory cell death pathway [20], and there are very few studies regarding the necroptosis in GBM. In our study, the ROC demonstrated that the gene profiling had high prediction ability in assessing the outcome of GBM at one, three, and five years.

In our single-cell-seq study, we first divided GBM cells into groups with high and low necroptosis score (Figure 1(d)), which provides us with a reference to explore the necroptosis heterogeneity in GBM. The tumor necrosis factor (TNF) is thought to be the initiating factor of necroptosis, by binding to tumor necrosis factor receptor 1 (TNFR1) on the plasma membrane, leading to formatting of necrosome by recruiting RIPK1 and RIPK3 [21]. It is worth noting that RIP1 autophosphorylation is facilitated by mitochondrial reactive oxygen species (ROS) and is critical for the recruitment of RIP3 into necrosome, which indicated that mitochondrial ROS promote necroptosis and the receptor interacting protein 1 (RIP1) is a key player in this form of cell death [22].

The pathway analysis in our study showed that cell cluster with higher inflammation shows high NCPTS. This validated that necroptosis is one kind of inflammation death. Accordingly, those cells with higher level of oxidative phosphorylation showed reduced NCPTS. We also found that the oxidative phosphorylation level was reduced in tumor-infiltrated cells including macrophages, T cells, and NK cells, while it was increased in the astrocytes [23]. This suggests that TNF and NFKB inflammation might induce the impaired oxidative phosphorylation in the immune cells. However, it is critical to explore the heterogeneity with different levels of inflammation and oxidative phosphorylation in tumor tissues and microenvironments.

Among the 10 prognosis-related necroptosis genes, we found that only NDUFB2 is highly increased in the glioma and statistically correlated with the overall survival in GBM. NADH: ubiquinone oxidoreductase (complex I) subunit B2 (NDUFB2) has NADH dehydrogenase activity and oxidoreductase activity. It plays an important role in transferring electrons from NADH to the respiratory chain. In addition, NDUFB2 has been reported to be associated with the pathogenesis of bipolar disease [24] and gram-negative sepsis [25]. However, the role of NDUFB2 in tumor, especially in GBM, is not extensively investigated. Our findings suggested that NDUFB2 is related to the poor outcome in GBM, and this result has been validated in CGGA as well.

To our knowledge, few reports regarding the necroptosis prognostic model in GBM with the single-cell cluster analysis have been previously reported. However, further experimental studies are warranted to validate these biomarkers and stratify patients to locate those with better benefits for effective personalized treatment [26].

## 5. Conclusion

A necroptosis gene profile related to survival status was identified in GBM. We also validated the effect of NDUFB2 in GBM by multiple datasets, which might provide clinical evidence for GBM interventions.

## Data Availability

The single-cell RNA sequencing data could be obtained from the corresponding author with a rational request. TCGA GBM data and GSE 43378 are publicly available from the official website.

## Supplementary Materials

Supplementary Materials Supp Figure 1: validated NDUFB2 expression in the CGGA dataset. (A) The expression of NDUFB2 in different grades of glioma. (B) The expression of NDUFB2 is much lower in the IDH-1 mutant type compared to IDH-1 wild-type groups. (C) The expression of NDUFB2 in four molecular types of glioma. (D) ROC in predicting NDUFB2 for CGGA subtype. (E) The protein-protein interaction of NDUFB2. (F) The distribution of NDUFB2 and NDUFB2-related genes in the high-grade glioma, classical subtype, and IDH-1 wild-type group. (G) The expression of NDUFB2 is negatively associated with the survival status in glioma. Supp Figure 2: the function of NDUFB2 has been investigated from CancerSEA web tool (http://biocc.hrbmu.edu.cn/CancerSEA). (A) NDUFB2 has a different role in LGG and glioblastoma. (B) NDUFB2 is related to the cell cycle, DNA damage, and DNA repair. (C) NDUFB2 is positively correlated with DNA damage, DNA repair, invasion, stemness, and cell cycle and negatively associated with inflammation and angiogenesis. Supp Figure 3: the function of NDUFB2 in U87 cells in vitro. (A) NDUFB2 promotes cell proliferation while siNDUFB2 reduces cell proliferation in U87 cell lines. (B) NDUFB2 promotes the proliferation and survival of glioma cell lines, *n* = 3, ∗/#, *P* < 0.05. The supplementary excel file is in https://www.jianguoyun.com/p/DYmqyDgQgoS1ChjIhMUEIAA.
