# Supplementary material for: Single-Cell Sequencing Reveals Necroptosis-Related Prognostic Genes of Glioblastoma
Source: Oxid Med Cell Longev. 2023 Feb 20;2023:2926655. doi: 10.1155/2023/2926655 (PMC9970716; doi:10.1155/2023/2926655)
Supplement: Supplementary Materials — Supp Figure 1: validated NDUFB2 expression in the CGGA dataset. (A) The expression of NDUFB2 in different grades of glioma. (B) The expression of NDUFB2 is much lower in the IDH-1 mutant type compared to IDH-1 wild-type groups. (C) The expression of NDUFB2 in four molecular types of glioma. (D) ROC in predicting NDUFB2 for CGGA subtype. (E) The protein-protein interaction of NDUFB2. (F) The distribution of NDUFB2 and NDUFB2-related genes in the high-grade glioma, classical subtype, and IDH-1 wild-type group. (G) The expression of NDUFB2 is negatively associated with the survival status in glioma. Supp Figure 2: the function of NDUFB2 has been investigated from CancerSEA web tool (http://biocc.hrbmu.edu.cn/CancerSEA). (A) NDUFB2 has a different role in LGG and glioblastoma. (B) NDUFB2 is related to the cell cycle, DNA damage, and DNA repair. (C) NDUFB2 is positively correlated with DNA damage, DNA repair, invasion, stemness, and cell cycle and negatively associated with inflammation and angiogenesis. Supp Figure 3: the function of NDUFB2 in U87 cells in vitro. (A) NDUFB2 promotes cell proliferation while siNDUFB2 reduces cell proliferation in U87 cell lines. (B) NDUFB2 promotes the proliferation and survival of glioma cell lines, n = 3, ∗/#, P < 0.05. The supplementary excel file is in https://www.jianguoyun.com/p/DYmqyDgQgoS1ChjIhMUEIAA. [file 2926655.f1.pdf]

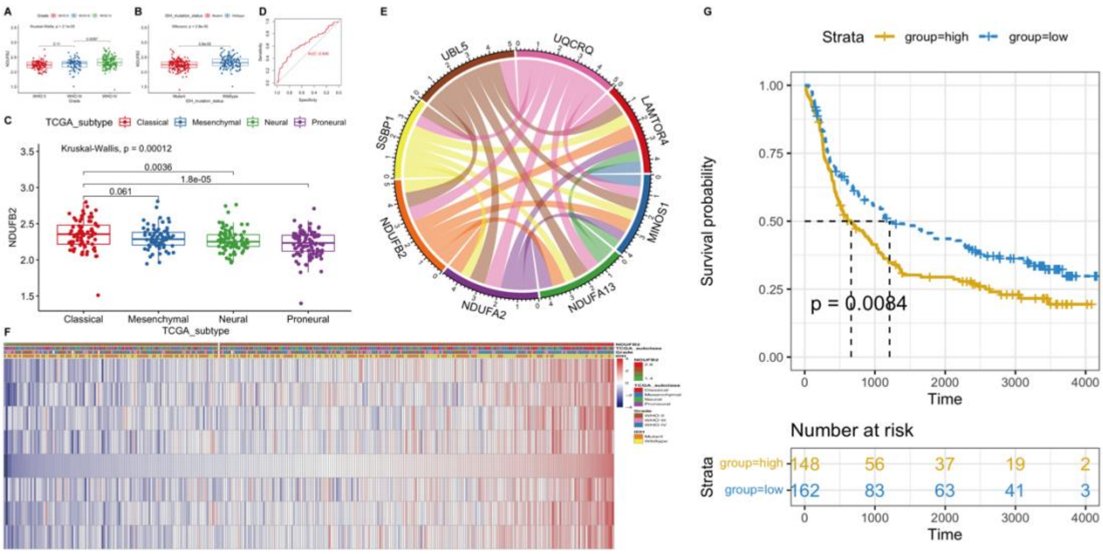

Supp Figure 1. Validated NDUFB2 expression in CGGA dataset. (A) The expression of NDUFB2 in different grades of glioma. (B) The expression of NDUFB2 is much lower in IDH-1 mutant type compared to IDH-1 wildtype groups. (C) The expression of NDUFB2 in four molecular types of glioma. (D) ROC in predicting NDUFB2 for CGGA subtype. (E) The protein-protein interaction of NDUFB2. (F) The distribution of NDUFB2 and NDUFB2-related genes in the high-grade glioma, classical subtype and IDH-1 wildtype group. (G) The expression of NDUFB2 is negatively associated with the survival status in glioma.

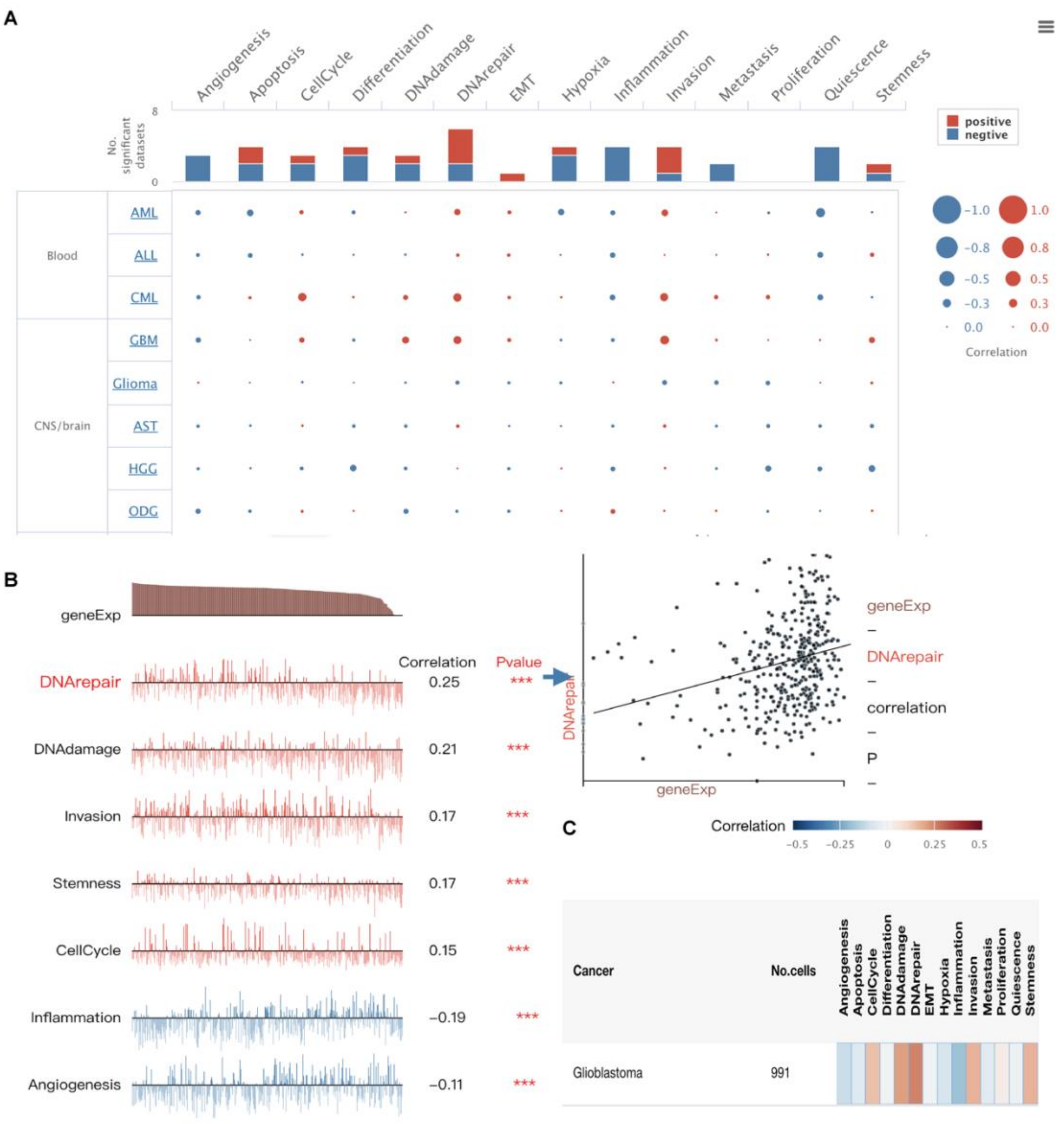

Supp Figure 2. The function of NDUFB2 has been investigated from CancerSEA web tool (biocc.hrbmu.edu.cn/CancerSEA)**.** (A) NDUFB2 has a different role in LGG and glioblastoma. (B) NDUFB2 is related to the cell cycle, DNA damage and DNA repair. (C) NDUFB2 is positively correlated with DNA damage, DNA repair, , invasion, stemness, cell cycle and negatively associated with inflammation and angiogenesis.

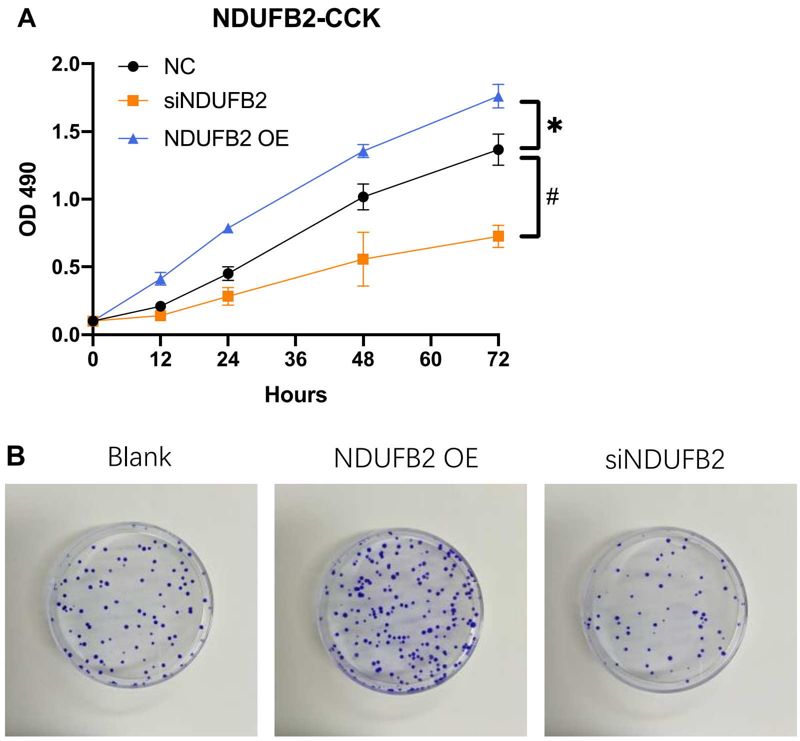

Supp Figure 3. The function of NDUFB2 in U87 cells in-vitro. (A) NDUFB2 promotes cell proliferation while siNDUFB2 reduces cell proliferation in U87 cell lines. (B) NDUFB2 promotes the proliferation and survival of glioma cell lines, n=3, */#, $p<0.05$.
